# Challenges and strategies in dental care for patients with intellectual disabilities in Hungary

**DOI:** 10.1186/s40902-025-00460-1

**Published:** 2025-03-21

**Authors:** Ilona Szmirnova, György Szmirnov, Emese Gellérd, Zsolt Németh, Márton Kivovics, György Szabó

**Affiliations:** 1 Department of Oro-Maxillofacial Surgery and Stomatology, Semmelweis University, Mária utca 52., 1085 Budapest, Hungary; 2 Department of Anesthesiology and Intensive Therapy, Semmelweis University, Üllői út 78., 1082 Budapest, Hungary; 3 Department of Public Dental Health, Semmelweis University, Szentkirályi u. 40., 1088 Budapest, Hungary

**Keywords:** Intellectual disability, Dental care, Preventive health services, Oral hygiene, Rehabilitation, Oral health

## Abstract

**Background:**

Providing adequate dental care and implementing preventive strategies for patients with intellectual disabilities (ID) pose significant challenges in Hungary, where approximately 100,000 individuals with ID require specialized dental care. This study aimed to objectively assess the dental and periodontal care needs of patients with ID in comparison to the general population and those with physical disabilities. Additionally, we developed and evaluated a program focusing on acute treatment and the prevention of dental diseases.

**Methods:**

A retrospective analysis was conducted over a 5-year period, involving the demographics and therapeutic outcomes of 1717 patients with ID who received dental care. Initial screening of dental status was performed for 350 patients with ID, and a structured preventive care program was developed and implemented for 49 patients.

**Results:**

Over the 5 years, 8147 dental interventions were performed under general anesthesia without major complications. Compared to the general population, patients with ID exhibited poorer Decayed and Missing scores but more favorable Filled scores based on the decayed, missing, and filled teeth (DMFT) index. The implementation of preventive measures led to significant improvements in periodontal health within 3–6 months.

**Conclusions:**

Despite the success of preventive measures, the overall therapeutic outcomes in patients with ID were suboptimal, with caries and periodontal diseases increasing with age and severity of disability. Structured oral hygiene programs are essential to improving the oral health of this vulnerable population.

## Background

Various challenges are encountered during the dental treatment of patients with intellectual disability, such as the relative increase in the number of these patients, the observation of human rights regarding healthcare success, and the improved level of care for intellectual disability, resulting in an increased need for dental care. Clinical and oral manifestations of intellectual disability are periodontal diseases; dental caries; structural abnormalities, such as growth disturbances and malocclusion; and destructive oral habits, such as bruxism and clenching.

According to the Hungarian Central Statistical Office, > 400,000 individuals had intellectual and physical disabilities in 2009 [1, 2]. Patients with severe intellectual disability present the single largest challenge during dental treatment as they can only be treated under general anesthesia. Notably, some patients with a mild or medium disability level can be treated with appropriate precautions under local anesthesia. During the past 8–10 years, methods for acute dental care for patients with severe intellectual disability have improved, and in major cities of Hungary, centers that provide dental surgical and periodontal treatments are available. Unfortunately, restorative, and prosthetic treatment can rarely be performed in patients with severe intellectual disability [3]. Dental treatment of patients with intellectual disability, assessment of their needs, and different preventive strategies have become a global concern. Surveys have been conducted in several countries regarding the number of patients requiring treatment, possible treatment and preventive options, and their results [4–15]. Patients with intellectual disabilities who cannot receive treatment under local anesthesia should instead be treated under general anesthesia [4–7]. For patients with intellectual disabilities, using antiplaque agents and treatments to reduce gingivitis is crucial for prevention [9–11, 14]. The deficit in oral hygiene for patients with intellectual disabilities is multifactorial, with risk factors including oral deformities and socioeconomic status [8, 11–13].

In the present study, we aimed to (1) retrospectively analyze the demographic data and therapeutic outcomes of patients with intellectual disability, including those requiring acute treatment, during 5 years at the Department of Oral and Maxillofacial Surgery at Semmelweis University (Budapest); (2) objectively assess the dental and periodontal needs of patients with intellectual disability in Hungary compared with those of the general population and patients with physical disability; and (3) and evaluate the effectiveness of a preventive training program developed for patients with intellectual disability.

## Methods

### Study design

A total of 1717 patients with intellectual disability have been treated under local and general anesthesia at the department from its opening, on October 1, 2014, up to December 31, 2018. The study was conducted at the Department of Oro-Maxillofacial Surgery and Stomatology, Faculty of Dentistry, Semmelweis University, and the Nursing Home and Daycare Institute of the Foundation for Equal Opportunities in Hungary. It was approved by the Medical Research Council, Hungary (ETT TUKEB IV/8158–3/2020/EKU) and conducted according to the ethical principles of the Helsinki Declaration. Acute treatment and screening data were collected retrospectively, whereas the influence of the preventive training program on the oral hygiene of patients with intellectual disability was evaluated prospectively. In the case of the prospective study, the procedures included were thoroughly explained to the participants and their guardians. Thereafter, all participants or their guardians provided written informed consent. Patients were classified into mild, medium, and severe stages of intellectual disability based on the assessment of a neurologist by the Diagnostic and Statistical Manual of Mental Disorders DSM-V [16]. Clinical trial number: not applicable. The screening was conducted at a nursing home, where all 325 residents were examined. Since all residents were screened, the process was not random. However, for preventive cases, a subset of 49 patients was randomly selected.

### Acute treatment

The epidemiological assessment of patients undergoing acute treatment was conducted on the entire patient population. Acute treatments were performed at the Department of Oro-Maxillofacial Surgery and Stomatology, Faculty of Dentistry, Semmelweis University. Patient history, types of interventions performed, and complications were obtained from the patients’ charts retrospectively. Preoperative evaluation could be performed only when the patient did not require treatment under general anesthesia. For technical reasons, radiographs could be obtained only for patients with a mild or medium level of disability. Before the induction of anesthesia, a telephonic consultation between the anesthetist and the patient's caretaker was conducted. Owing to the non-ambulatory nature of patients with severe disability, general, cardiologic, and neurologic evaluations were performed at the discretion of the anesthetist, based on the telephonic consultation.

### Intravenous anesthesia

General anesthesia was induced using 2 mg midazolam (Midazolam Accord 5 mg/mL, Solution for Injection or Infusion; Accord Healthcare, Pabianice, Poland) and 0.5–1 mg/kg propofol (Propofol-Lipuro 1% [10 mg/mL] emulsion for injection or infusion; B. Braun Melsungen, Melsungen, Germany); a 20–30 mg bolus of propofol was added if needed. Then, oxygen was administered at 2 L/min through a nasal probe. At the end of the intervention, the effect of midazolam was reversed using 0.2 mg flumazenil (Flumazenil 0,1 mg/ml; Pharmaselect International Beteiligungs GmbH, Wien, Austria).

### Inhalational anesthesia

Following the administration of 2 mg midazolam, 50–100 mg fentanyl (Fentanyl Kalceks 50 µg/mL solution for injection; AS Kalceks, Riga, Latvia), 1–1.5 mg propofol, and 0.5 mg/kg atracurium (Atracurium Besilate Kalceks 10 mg/mL solution for injection/infusion; AS Kalceks) were administered through intubation. Narcosis was maintained using a Sevorane inhaler (Sevoflurane Inhalation Anaesthetic Solution, 250 mL; AbbVie Inc., North Chicago, IL, USA) with 50% oxygen and 50% nitrous oxide. At the end of the intervention, the effect of midazolam was reversed using 0.2 mg flumazenil. Following the operation and reversal of anesthesia, patients were inspected by the personnel of the rehabilitation center [15]. Notably, most patients were scheduled for regular follow-up visits (every 3–6 months); in case of any difficulty, the head of the center liaised with the patient’s caretaker.

### Screening

The screening was conducted at a nursing home, where all 325 residents were examined. To evaluate the effectiveness of the devised training program, a subset of 49 patients was randomly selected. Caries assessment was performed. A total of 325 patients were categorized into three groups: mild (*n* = 36), medium (*n* = 247), and severe (*n* = 42) intellectual disability. Decayed (D), missing due to caries (M), and filled (F) teeth in the permanent teeth (DMF-T) index was used to assess the patients’ dental health status according to the World Health Organization guidelines, and the results were compared with those of patients with physical disability and the general population [17, 18].

### Prevention

A group of 49 patients was randomly chosen from nursing home residents and participated in the preventive training program that we developed. For precise data collection, each patient was examined separately by three different doctors. First, patients and caretakers were trained in dental care. The assessment and management of the periodontal condition were performed by the team’s periodontologist using the Basic Periodontal Examination (BPE). Patients were followed up, and data was collected after 3–6 months. The training program began with an educational session; the conventional oral hygiene training program was modified according to the patient’s intellectual condition. The complex task of cleaning the teeth was simplified into separate steps to enable patients to follow instructions easily. Each patient was provided a toothbrush and toothpaste. The patients were taught the method of cleaning the chewing surfaces using a toothbrush in the first session, as well as other surfaces in subsequent sessions. It is important to have short sessions and regularly emphasize the instructions, as aided by the caretakers [17]. The patients were given the opportunity to provide feedback during each meeting. This was followed by immediate and positive encouragement (a smile or a gift) from the caretakers. We attempted to gain the cooperation of the patients throughout the 1-week training period. The properly-trained caretakers provided dental care to the patients with severe disability. Instructions were provided to the patients to rinse their teeth after each meal, which the caretakers ensured [19].

### Statistical analysis

Data was collected from the general population (4606 individuals; 2923 women and 1683 men) who underwent mandatory lung screening examinations [20]. The population with physical disabilities (608 patients) was randomly selected from the National Institute of Medical Rehabilitation [21].

We calculated the mean and standard deviation of patients’ D, M, F, and DMF-T scores and compared these values with those of the general population, adjusted for sex and age, using a two-sample *t*-test. Additionally, a two-sample t-test was employed to compare the scores of patients living in family settings with those residing in institutions. The relationship between the D, M, F, and DMF-T scores and the severity of intellectual disability was assessed using one-way analysis of variance (ANOVA).

To evaluate the effectiveness of the training program, BPE scores from different examinations (baseline, 3-month control, and 6-month control) were compared using a paired-sample t-test.

A *P*-value of < 0.05 was considered statistically significant. All statistical analyses were conducted using SPSS Statistics version 25.0 (IBM Corporation, Armonk, NY, USA) [17].

## Results

### Acute treatment

The most important finding of the present study was that considerable progress was achieved in the field of acute dental care for patients with intellectual disability. Table 1 summarizes the distribution of patients by diagnosis and age. The majority of patients presented with intellectual disability: 25 with a mild, 695 with a medium, and 326 with a severe level of intellectual disability. Of the patients included in the present study, 185 had autism spectrum disorders. Of the patients with Down syndrome, 101 and 32 had mild and severe levels of intellectual disability, respectively.

**Table 1:** Breakdown of patients according to diagnosis

Condition	No. of patients	Male	Female	Average age (years)
Mild level of intellectual disability	125	84	41	30
Medium level of intellectual disability	695	393	302	36
Serious level of intellectual disability	326	284	42	28
Down syndrome mild	101	80	21	33
Down syndrome serious	32	18	14	27
Autism spectrum disorder	185	95	90	29
Asperger’s syndrome	36	22	14	29
Hallervorden–Spatz syndrome	1	1	-	31
Sclerosis tuberosa	2	2	-	19
Fragilis X syndrome	1	1	-	21
Williams–Beuren syndrome	3	3	-	25
Beckwith–Wiedemann syndrome	1	-	1	21
Epilepsy	166	124	42	37
Panic disorder	43	8	35	32
Total	**1,717**	**1,115**	**606**	**32.8**

Patients with epilepsy (*n* = 166) and panic disease (*n* = 43) were treated similarly to those with intellectual disability, under general anesthesia only. Among the patients with other syndromes, 36 had Asperger’s syndrome, one had Hallervorden–Spatz disease, two had sclerosis tuberosa, one had fragile X syndrome, one had Beckwith–Weidemann syndrome, and three had Williams–Beuren syndrome. Table 1 presents the average age of each patient group. Of the included patients, 1115 were male, whereas 602 were female, with an average age of 32.8 years.

Table 2 presents the type of interventions performed. Notably, most interventions were extraction and surgical extraction (4219 and 1691 patients, respectively), which were acute interventions. Restorations were performed either simultaneously with the acute interventions or at a different point in time, under anesthesia. Whenever possible, efforts were made to restore all carious teeth. Therefore, 2616 restorations were performed in 1610 patients at the time of acute treatment. Notably, most patients had compromised dental hygiene; therefore, scaling was performed in most patients (*n* = 1184). Cystectomy (10 radicular and two follicular) was performed in 12 patients. The outcomes of 12 biopsies were 10 inflamed lesions and two epulis (peripheral giant cell granuloma). Endodontic treatment (*n* = 104) of the anterior teeth was performed in 87 patients, mostly for pulpitis.

**Table 2:** Type of acute intervention

Intervention	No. of interventions
Filling	2616
Extraction, sculption	4219
Root canal filling	104
Cystectomy	12
Scaling and root planing	1184
Biopsy	12
Total	**8147**

### Complications arising during either dental treatment or anesthesia

During surgery, the most frequent complication (*n* = 107) was the fracture of teeth or roots. No case of hemorrhage that could not be addressed by sutures occurred. Postoperative hemorrhage during the observation period occurred in 12 cases, which were managed under another session of anesthesia. Postoperative inflammation (alveolitis) occurred in 41 cases. Notably, most of these cases were managed using antibiotics and non-steroid anti-inflammatory drugs; only eight patients required further surgical intervention (enucleation or extraction of endodontically treated tooth). Of the 2616 restorations, pulpitis occurred in 47, prompting extraction. During anesthesia, five patients had desaturation, whereas 18 had agitation. Emesis or nausea after anesthesia was observed in 80 patients, and fever with chills was observed in 11 patients. No case of nose hemorrhage occurred because the intratracheal tube was not inserted through the nose.

### Screening

**Fig. 1:**
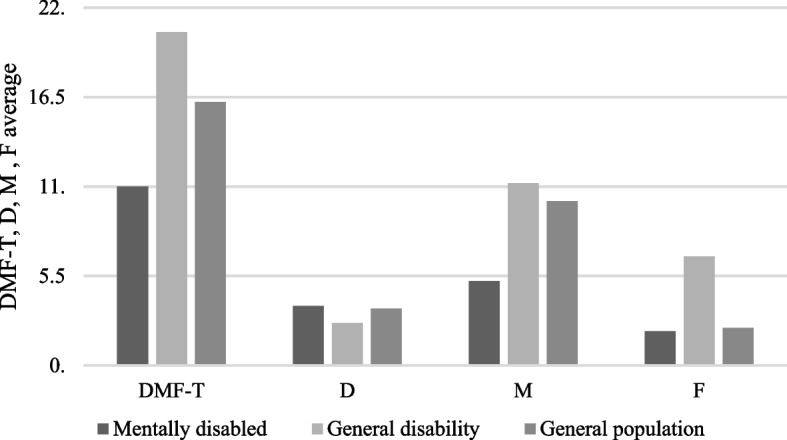
Dental status of patients with intellectual disability, patients with general disability, and the general population. Dental status of patients with intellectual disability (*n* = 326); patients with general disability (*n* = 608), and general population (*n* = 4,606). DMF-T = decayed, missing, filled tooth

The mean DMF-T score of the 325 patients with intellectual disability was 11.04. The mean number of decayed teeth, “D,” was 3.66, whereas that of missing teeth, “M,” was 5.22. Minimum evidence of restorative dentistry was observed. The mean number of filled teeth, “F,” was 2.16 (Table 3). The more severe the intellectual disability, the poorer the patient’s dental status. In patients with a severe level of intellectual disability, the mean number of decayed teeth was significantly higher (mild, *D* = 3.19; medium, *D* = 3.41; severe, *D* = 5.52; *P* = 0.0184). This was confirmed by one-way ANOVA at *P* < 0.05, indicating that the mean values were significantly different depending on the disability level. Figure 1 presents the dental status of patients with intellectual disability, patients with general disability [21], and the general population [20]. Furthermore, at a younger age, the number of decayed teeth was higher, whereas the number of extracted teeth increased with increasing age. Additionally, the dental condition of patients living in a family was significantly superior to that of those living in an institution (DMF-T score, 9.76 vs. 12.39, *P* = 0.0013) (Table 4).

**Table 3:** Average D, M, and F indices and ± variance of patients with intellectual disability

	***n*** ** (%)**	**DMF-T**	**D**	**M**	**F**
**Complete sample**	325 (100%)	11.04	3.66	5.22	2.16
**Mild**	36 (11.1%)	11.00	3.19	5.17	2.64
**Medium**	247 (76.0%)	10.72	3.41	5.24	2.07
**Severe**	42 (12.9%)	12.98	5.52	5.14	2.31
**ANOVA *P*- value**	-	0.1849	0.0184*	0.9934	0.5627

^*^One sample variance analysis (ANOVA) *P* < 0.05 indicates that average values are significantly different depending on the level of disability

*DMF-T *Decayed, missing, filled tooth, *ANOVA *Analysis of variance

**Table 4:** Dental status of patients living in a family environment or being institutionalized

	**Patients living in a family environment (*n* = 171)**		**Institutionalized patients (*n*Institutionalized patients = 154)**		**Two sample *t*-test *P*-value**
	Average	Variance	Average	Variance	
**D**	3.16	4.26	4.22	4.92	0.0392*
**M**	4.33	4.49	6.21	6.74	0.0037*
**F**	2.27	3.09	2.04	3.16	0.4970
**DMF-T**	9.76	6.76	12.39	7.77	0.0013*

^*^Two-sample *t*-test *P* < 0.05 indicates that, except for filled teeth, there is a significant advantage regarding dental status in favor of patients living in a family environment

*DMF-T *Decayed, missing, filled tooth

### Prevention

The most important result of the present study was the successful use of our preventive program at the Nursing Home and Daycare Institute of the Foundation for Equal Opportunities in Hungary. This indicated that the simplified training program, introduced to 49 examined patients, showed significant results. Regarding the periodontal status of the patient in the training program, the majority of the patients fell into Stage III and IV, Grade B and C. This conclusion was drawn based on clinical findings where extensive tooth loss and furcation involvement of multi-rooted teeth were observed.

The first and second control examinations (3 and 6 months, respectively) confirmed that patients showed a statistically significant improvement in the periodontal index compared with the baseline values (baseline, 17.69; control 1 = 15.86; control 2 = 14.45).

The following results were obtained depending on the disability level. In patients with a mild level of intellectual disability, when comparing the baseline and control 2 values, the improvement in the periodontal index scores was significant (*n* = 33; baseline, 17.12; control 1 = 15.48; control 2 = 14.35). In patients with a medium level of disability, the improvement in the periodontal index scores was significant after controls 1 and 2 (*n* = 12) compared with that at baseline (baseline, 19.5; control 1 = 16.75; control 2 = 13.73). In patients with a severe level of disability, the periodontal index scores were not significantly different between the baseline and control examinations (*n* = 4; baseline, 17.1; control 1 = 16.25; control 2 = 17.25) (Table 5.). Patient education can be helpful to patients with a mild or medium level of disability. However, considering the limited number of patients with a severe level of disability, the results were not statistically significant. Nonetheless, patient education was useful for all three groups.

**Table 5:** Changes in the periodontal index at 3 and 6 months in patients based on the division by sex and age group

	***n***	**Starting point**	**1. control**	**2. control**	**Level of significance**
		Mean	Mean	Mean	
Complete sample	49	17.69	15.86	14.46	*P* < 0.000*
Mild level of intellectual disability	33	17.12	15.48	14.35	*P* < 0.000*
Medium level of intellectual disability	12	19.5	16.75	13.73	*P* = 0.010*
Severe level of intellectual disability	4	17	16.25	17.25	*P* = 0.368

^*^Paired-samples *t*-test *P* < 0.05. The asterisk indicates that there is a significant difference between the values

## Discussion

The results of the present study reveal the difficulties and the necessity of the dental treatment of intellectually disabled patients. Acute treatment is costly as separate rooms, dentists, and general anesthesia are needed to perform it. That is, such treatment is more costly and more difficult to carry out, than the treatment of the general population. According to surveys, the more serious the condition and the higher the age of the patients, the more decayed teeth they will have. In view of the above, it is clear that prevention, which can be successful based on proper education, is of utmost importance. Prevention, therefore, should reach out to a broader circle of patients.

Based on the surveys conducted on the treatment of patients with intellectual disability, there is an agreement in the literature regarding the kind of anesthesia required. Patients with intellectual disabilities who cannot receive treatment under local anesthesia should instead be treated under general anesthesia [4–7]. The literature emphasizes the benefits of antiplaque agents and professional hygiene treatments to reduce gingivitis and supports that this is crucial for the prevention of oral diseases [9–11, 14]. In the case of patients with intellectual disabilities, the deficit in oral hygiene cannot be explained solely by the lower motivation and capability to maintain sufficient oral hygiene. Risk factors such as the caretaker’s lack of motivation and knowledge in delivering oral hygiene, oral deformities, and socioeconomic status play an important role in the oral hygiene deficit of this patient group of special needs [8, 11–13].

In Hungary, approximately 100,000 patients have intellectual disability, with dental treatment being administered to them under general anesthesia only. Within the 5-year study period, approximately 1800 patients required acute treatment in Budapest. Regarding patients with intellectual disability, the provision of acute interventions primarily under general anesthesia has been proposed [5, 6]. While evaluating the findings related to Semmelweis University, we discovered that similar results were evident in another center dedicated to treating patients with intellectual disabilities in Hungary, albeit with a smaller patient population [22].

Notably, some dentists aim for full-mouth rehabilitation (extraction, restorations, and dentures) during general anesthesia. However, this results in a longer duration of anesthesia and a lower number of patients being treated (Solanki et al. [7] reported 200 cases in 10 years). Furthermore, these patients should be removed from their usual surroundings for a minimal period [5, 6, 23, 24]. Additionally, intraoperative, and postoperative complications related to anesthesia should be considered. Despite the significantly higher number of interventions in our study, no patient had nose hemorrhage because the intratracheal tube was not inserted through the nose. Only a few cases of desaturation and emesis were observed.

Of the 2000 patients treated at our institution, 90% required further restorative or prosthetic treatment. The prevalence of caries in the standard population in Hungary [2] is higher than that of most European populations (DMF-T score: 3.3 in Hungary [20, 25–28] vs. 0.8 in the Netherlands [29]). Comparing the dental status of the standard population with that of patients with intellectual disability who have a high DMF-T score of 3.3 would ultimately produce an even poorer comparative score. Notably, no survey regarding the dental status of patients with intellectual disability has been published in a Hungarian scientific journal, although this could provide objective data. However, several studies worldwide have compared the dental status, relevant anatomical formulas, jawbones, and temporomandibular joints between these patients [30–33].

Iszmailov [13] examined 171 patients with intellectual disability and reported that 34% of the patients develop periodontal problems at the age of 18–25 years, whereas 82% develop periodontal problems after the age of 42 years. Mac Giolla Phadraig et al. [34], in the framework of the “Delphi panel,” determined the dental care that could be performed in patients with intellectual disability. They highlighted 16 consensual findings regarding personalized treatment, information flow, training, and costs. The most important findings referred to the buildings, equipment, and personnel involved in providing treatment. Notably, some countries have demonstrated limited progress. In an initial step, Waldman et al. [35] determined that the nationwide need for dental treatment of patients with intellectual disability should be established based on research and followed by the establishment of centers where dental treatment (including restorative dentistry and prevention), excluding the treatment by general practitioners and psychiatrists, would be available.

The number of patients requiring more than acute care exceeds the available capacity (institutions, dentists, and financial resources). Therefore, prevention should be prioritized. Notably, several prevention methods have been documented, with the results reported by Edwards et al. [8] in 2002 being the most significant. In Merseyside County (Liverpool area, mid-West England), dentists were invited to provide treatment for patients with intellectual disability. Training programs were organized for patients and their relatives to improve oral hygiene. These programs resulted in a considerable decrease in the number of patients requiring acute dental treatment and an improvement in the dental status of patients [36, 37]. Even though acute dental treatment modalities for patients with intellectual disability have recently improved in Hungary (approximately 3,000 patients were treated at five centers during the last 5 years), opportunities for complex dental rehabilitation remain limited [2].

Our screening research included > 300 patients, consistent with the sample size in previous studies: 225 in India [36] and 221 in Germany [38, 39]. We originally aimed to compare the dental status of patients with intellectual disability with that of the general population; however, we also compared our findings with data pertaining to individuals with a general (mainly physical) disability (Fig. 1) [39–42]. The mean DMF-T score of the general population (4606 examined patients) was 16.2, whereas that of patients with a general disability (608 patients) was 20.5. In 325 patients with intellectual disability, the DMF-T score was 11. When comparing the dental status of patients with intellectual and physical disabilities and the general population, we found that patients with intellectual disability had the highest number of decayed teeth and the lowest number of filled and missing teeth [20, 21].

We compared the dental status of patients with intellectual and physical disabilities and the general population in an age group of 20–44 years because 85% of the examined patients were in this age group. For patients with intellectual disability and those in the general population aged ≤ 19 years, the mean (± standard deviation) DMF-T score was 6.09 ± 7.13 and 11.24 ± 4.85, respectively; in the age group of 20–34 years, it was 9.21 ± 6.33 and 12.76 ± 5.45, respectively; and in the age group of 35–44 years, it was 11.91 ± 6.77 and 15.40 ± 5.13, respectively. To understand this apparent paradox, we need to simplify the DMF-T score. Patients with intellectual disability had the highest number of decayed teeth (D = 3.7). Providing dental treatment to such patients is complicated because most require general anesthesia; therefore, the most frequent intervention is extraction (*M* = 5.2). Restorative treatment is rarely performed (*F* = 2.2). In the general population, F, D (lowest), and M were 2.3, 3.5, and 10.1, respectively. In patients with general disabilities, M was 11.2.

Providing dental treatment to patients with intellectual disability is challenging. Therefore, preventive measures should be applied as widely as possible. In the United States, treatment of patients with special needs (including those with intellectual disability) is a part of the curriculum at several universities [43]. Comparing the toothbrushing habits of healthy children and those with intellectual disability of the same age [44], patients with intellectual disability pay less attention to their teeth than healthy children. Therefore, in terms of prevention, patients with intellectual disability need enhanced care. In the United States [45], children with intellectual disability (those born in the 1990s) were examined and educated in the presence of their parents before school about the importance of cleaning their teeth and maintaining oral hygiene. Follow-up examinations performed subsequently showed that the dental condition of these children was considerably better than that of other children needing special care.

The survey by Boyle et al. [46] indicates that the prevalence of patients with intellectual disability in the United States is approximately 6.7/1000. Due to poor oral hygiene, 90% of these patients develop periodontal diseases [47]. According to Wyne [48] in a study in Pakistan, most patients with intellectual disability have periodontal diseases because of inadequate oral hygiene and not due to their basic condition. Notably, Hungarian authors [49, 50] have reported the general and dental conditions of patients with physical disability and have recommended the implementation of special programs accordingly [51–54].

A limitation of this study was the small sample size enrolled in the preventive care program which limits the generalizability of its findings. However, the sample size was sufficient to prove the feasibility of the preventive program for patients with intellectual disability. A further limitation regarding the comparison of the DMF-T scores of the intellectually disabled patients to that of the general population and population with general disability was that a multivariate analysis could have accounted for some confounding factors (e.g.: socioeconomic status or access to care).

The findings of control examinations performed after 3 or 6 months did not reveal any apparent improvement in the present study. This was not unexpected, as the interval of 3–6 months is too short to observe any progress in the reduction of caries incidence. Relying on our proposed relatively simple training program, we showed that the scope of our work should be widened. Improved dental care and enhanced (normal) oral hygiene maintenance can result in substantial changes in a relatively short period. The attitude of the caretakers and parents toward the program is important. The first phase of rehabilitation is the survey of needs; the second is the implementation of prevention procedures; and the third, which is the most difficult and expensive, is performing adequate surgical and/or restorative treatments. The first phase is complete, whereas the second, as reflected by the current prevention program, is underway. The third phase, as far as acute treatments are concerned, is partially complete; however, in the field of restorative dentistry, limited progress is evident, with prevention programs playing a significant role.

The retrospective study results on acute treatments for patients with intellectual disabilities indicate that it is crucial to enhance funding for facilities offering this type of treatment and to establish more centers. Furthermore, it is crucial to implement preventive training programs in nursing homes to improve the oral hygiene of patients with intellectual disabilities and reduce the need for invasive surgery under general anesthesia.

## Conclusions

Based on our study and global findings, we conclude the following:

1. The incidence of caries and periodontal diseases increases with both age and the severity of intellectual disability, underscoring the need for proactive dental care.
2. Oral hygiene remains unsatisfactory across all levels of intellectual disability, necessitating targeted interventions.
3. Patients with intellectual disabilities and their caretakers lack sufficient knowledge about oral hygiene maintenance. Few participate in proper training, and motivation to maintain oral hygiene is generally low.
4. Routine dental treatments, aside from acute care, are inadequately provided, further exacerbating oral health disparities in this population.
5. Personalized oral hygiene instructions, the introduction of fluoride-containing toothpaste and mouthwashes, and enhanced training and motivation for caretakers could significantly improve oral health outcomes.
6. Acute dental care and full-mouth rehabilitation play a crucial role in maintaining masticatory function and preventing complications that might necessitate more invasive interventions.

From a maxillofacial surgery perspective, the oral health challenges faced by patients with intellectual disabilities can lead to severe outcomes, including advanced periodontal diseases and extensive tooth loss, which may require surgical interventions such as extractions, alveoloplasty, or pre-prosthetic surgeries under general anesthesia. Furthermore, the management of facial infections and complications arising from untreated dental issues often requires multidisciplinary care involving maxillofacial surgeons. Given the limited resources, particularly anesthesia facilities, achieving widespread improvement in oral health remains challenging. Therefore, as supported by previous studies, we emphasize the need for preventive strategies not only to reduce the incidence of acute interventions but also to limit the progression of oral diseases that necessitate maxillofacial surgical interventions. Strengthening the integration of preventive care with surgical expertise could enhance outcomes and optimize resource utilization.

## Data Availability

The datasets used and/or analyzed during the current study are available from the corresponding author on reasonable request. Open access funding provided by Semmelweis University.

## Abbreviation

DMF-T: Decayed (D), Missing due to caries (M), and Filled (F) Teeth in the permanent dentition

## References

[1] Representative Information Service. [Info Note 2013/13: About persons with disabilities] [Hungarian]. http://www.parlament.hu/documents/10181/59569/Infojegyzet_2013_13_fogyatekossaggal_elok.pdf/f5e90323-1f76-455f-83ca-4ae87239349f. Accessed 15 Apr 2013

[2] Central Statistical Office. [Disability and health status ageing due to population character (2016)] [Hungarian]. https://www.ksh.hu/docs/hun/xftp/idoszaki/mikrocenzus2016/mikrocenzus.2016 8.pdf. Accessed 15 Sep 2021

[3] Ajami BA, Shabzendedar M, Rezay YA, Asgary M (2007) Dental treatment needs of children with disabilities. J Dent Res Dent Clin Dent Prospects 1:93–98 23277841 10.5681/joddd.2007.016 PMC3525932

[4] Binkley CJ, Johnson KW, Abadi M et al (2014) Improving the oral health of residents with intellectual and developmental disabilities: an oral health strategy and pilot study. Eval Program Plann 47:54–63 25137553 10.1016/j.evalprogplan.2014.07.003 PMC4188479

[5] McKelvey VA, Morgaine KC, Thomson WM (2014) Adults with intellectual disability: a mixed-methods investigation of their experiences of dental treatment under general anaesthetic. N Z Dent J 110:58–64 25000808

[6] Wang Y-C, Huang G-F, Cheng Y-J et al (2015) Analysis of clinical characteristics, dental treatment performed, and postoperative complications of 200 patients treated under general anesthesia in a special needs dental clinic in northern Taiwan. J Dent Sci 10:172–175

[7] Solanki N, Kumar A, Awasthi N, Kundu A, Mathur S, Bidhumadhav S (2016) Assessment of oral status in pediatric patients with special health care needs receiving dental rehabilitation procedures under general anesthesia: a retrospective analysis. J Contemp Dent Pract 7:476–479 10.5005/jp-journals-10024-1875 27484601

[8] Edwards DM, Merry AJ, Pealing R (2002) Disability part 3: improving access to dental practices in Merseyside. Br Dent J 193:317–319 12368887 10.1038/sj.bdj.4801554

[9] Montiel-Company JM, Almerich-Silla JM (2002) Efficacy of two antiplaque and antigingivitis treatments in a group of young mentally retarded patients. Med Oral 7:136–143 11887021

[10] Reuland-Bosma W (2013) [Dissertations 25 years after date 35. Periodontal disease in Down syndrome: an immunological disorder] [Dutch]. Ned Tijdschr Tandheelkd 120:541–545 25026741

[11] Solanki J, Khetan J, Gupta S, Tomar D, Singh M (2015) Oral rehabilitation and management of mentally retarded. J Clin Diagn Res 9:ZE01-6 25738098 10.7860/JCDR/2015/11077.5415 PMC4347189

[12] Chhajed S, Bhambhani G, Agarwal R, Balsaraf S (2016) Impact of various extra-oral factors on caries experience among mentally disabled children residing in Bhopal city, central India: a cross-sectional study. J Indian Soc Pedod Prev Dent 34:285–290 27461815 10.4103/0970-4388.186744

[13] Iszmailov AI (2008) [Prophylaxis of oral diseases in patients with disabilities] [Russian]. Thesis. Russian University of Medicine (ROSUNIMED), Moscow (http://medical-diss.com/docreader/553130/a?#?page=6). Accessed 6 Nov 2008

[14] Chang J, Lee JH, Son HH, Kim H-Y (2014) Caries risk profile of Korean dental patients with severe intellectual disabilities. Spec Care Dentist 34:201–207 25039380 10.1111/scd.12047

[15] Szmirnova I, Gellérd E, Pintér GT, Szmirnov G, Németh Z, Szabó G (2019) [Dental and dental oralsurgical treatment of the mentally retarded in Hungary: the situation in the past, currently and hopes for the future] [Hungarian]. Orv Hetil 160:1380–1386 31448643 10.1556/650.2019.31475

[16] American Psychiatric Association (2013) Diagnostic and statistical manual of mental disorders (5th ed.). 10.1176/appi.books.9780890425596

[17] PE Petersen, RJ Baez, O (2013) World Health, Oral health surveys: basic methods, 5th ed., World Health Organization, Geneva

[18] Szmirnova I, Szmirnov G, Rencz F et al (2021) [Dental survey of the mentally disabled patients] [Hungarian]. Orv Hetil 162:1698–1702 34657002 10.1556/650.2021.32215

[19] Szmirnova I, Szmirnov G, Haba N, Csomó K, Németh Z, Szabó G (2023) [Dental care and prevention possibilities for the mentally disabled currently] [Hungarian]. Orv Hetil 164:1456–1461 37717238 10.1556/650.2023.32853

[20] Madléna M, Hermann P, Jáhn M, Fejérdy P (2008) Caries prevalence and tooth loss in Hungarian adult population: results of a national survey. BMC Public Health 21:364 10.1186/1471-2458-8-364 PMC2596128 18939981

[21] Orsós M, Moldvai J, Kivovics P, Németh O (2018) Oral health related quality of life of patients undergoing physical medicine and rehabilitation. Orv Hetil 159:2202–2206 30582355 10.1556/650.2018.31202

[22] Faculty of Dentistry, University of Szeged (2025) [Announcement regarding the management of patients with an intellectual disability] [updated 2025/01/24. Available from: https://www.stoma.u-szeged.hu/hirek-esemenyek/2015-november/fogyatekkal-elo?folderID=35217&objectParentFolderId=31203

[23] Wang YC, Lin IH, Huang CH, Fan S-Z (2012) Dental anesthesia for patients with special needs. Acta Anaesthesiol Taiwan 50:122–125 23026171 10.1016/j.aat.2012.08.009

[24] Maeda S, Tomoyasu Y, Higuchi H, Mori T, Egusa M, Miyawaki T (2012) Midazolam is associated with delay in recovery and agitation after ambulatory general anesthesia for dental treatment in patients with disabilities: a retrospective cohort study. J Oral Maxillofac Surg 70:1315–1320 22381698 10.1016/j.joms.2012.01.004

[25] Sitkin SI, Gasparian AL, Iu Ivanova T, Iu Nesterova E, Drozdova NI (2015) [Long-term dental interventions in mentally retarded children under general anesthesia with sevoflurane] [Russian]. Stomatologiia (Mosk) 94:59–60 25909619 10.17116/stomat201594159-60

[26] Károlyházy K, Schmidt P, Bogdán S, Hermann P, Arányi Z (2014) [Prosthodontic treatment of an edentulous epileptic patient with an implant-retained overdenture] [Hungarian]. A case report. Ideggyogy Sz 67:342–346 25518263

[27] Szanto I, Sandor B (2014) [Preventive health care of patients with special needs] [Hungarian]. Magy Fog 5:256–258

[28] Kállay HZ (2018) The aims and effects of conductive education, i.e. the process of developing orthofunction] [Hungarian]. Különleges Bánásmód 4:73–92

[29] Schuller AA, van Dommelen P, Poorterman JH (2014) Trends in oral health in young people in the Netherlands over the past 20 years: a study in a changing context. Community Dent Oral Epidemiol 42:178–184 24635669 10.1111/cdoe.12070

[30] Tanboga I, Durhan MA, Durmus B, Marks LA (2014) Temporomandibular disorders in young people with an intellectual disability: prevalence of signs and symptoms. Eur J Paediatr Dent 15:349–354 25517578

[31] Abeleira MT, Outumuro M, Ramos I, Limeres J, Diniz M, Diz P (2014) Dimensions of central incisors, canines, and first molars in subjects with Down syndrome measured on cone-beam computed tomographs. Am J Orthod Dentofacial Orthop 146:765–775 25432258 10.1016/j.ajodo.2014.08.016

[32] McKinney CM, Nelson T, Scott JM, Heaton LJ, Vaughn MG, Lewis CW (2014) Predictors of unmet dental need in children with autism spectrum disorder: results from a national sample. Acad Pediatr 14:624–631 25439161 10.1016/j.acap.2014.06.023 PMC4367192

[33] Naouri D, Bussiere C, Pelletier-Fleury N (2018) What are the determinants of dental care expenditures in institutions for adults with disabilities? Findings from a national survey. Arch Phys Med Rehabil 99:1471–1478 29355507 10.1016/j.apmr.2017.12.018

[34] Mac Giolla Phadraig C, Nunn J, Dougall A, O’Neill E, McLoughlin J, Guerin S (2014) What should dental services for people with disabilities be like? Results of an Irish Delphi panel survey. PLoS One 9:e113393 25420015 10.1371/journal.pone.0113393 PMC4242628

[35] Waldman H, Perlman S, Swerdloff M (2001) Children with mental retardation/developmental disabilities: do physicians ever consider needed dental care? Ment Retard 39:53–56 11270214 10.1352/0047-6765(2001)039<0053:CWMRDD>2.0.CO;2

[36] Viana GR, Teiltelbaum AP, dos Santos FA, Sabbagh-Haddad A, Guaré RO (2014) Chlorhexidine spray as an adjunct in the control of dental biofilm in children with special needs. Spec Care Dentist 34:286–290 25353657 10.1111/scd.12069

[37] Sinha N, Singh B, Chhabra KG, Patil S (2015) Comparison of oral health status between children with cerebral palsy and normal children in India: a case-control study. J Indian Soc Periodontol 19:78–82 25810598 10.4103/0972-124X.145800 PMC4365163

[38] Schulte AG, Freyer K, Bissar A (2013) Caries experience and treatment need in adult with intellectual disabilities in two German regions. Community Dent Health 30:39–44 23550506

[39] Schiffner U (2006) Krankheits- und Versorgungsprävalenzen bei Erwachsenen (35–44 Jahre). In: Micheelis W, Schiffner U (eds). Vierte Deutsche Mundgesundheitsstudie (DMS IV). Institut der Deutschen Zahnärzte, Köln, pp 241–265

[40] Körmöczi K, Komlós G, Papócsi P, Horváth F, Joób-Fancsaly Á (2021) The early loading of different surface-modified implants: a randomized clinical trial. BMC Oral Health 21:207 33902551 10.1186/s12903-021-01498-z PMC8074492

[41] Joób-Fancsaly Á, Karacs A, Pető G, Körmöczi K, Bogdán S, Huszár T (2016) Effects of a nano-structured surface layer on titanium implants for osteoblast proliferation activity. Acta Polytech Hungarica 13:7–25

[42] Kaposvári I, Körmöczi K, Csurgay K et al (2021) Delayed-onset infections after lower third molar surgery: a Hungarian case-control study. Oral Surg Oral Med Oral Pathol Oral Radiol 132:641–647 34518142 10.1016/j.oooo.2021.04.052

[43] Dehaitem MJ, Ridley K, Kerschbaum WE, Rohr IM (2008) Dental hygiene education about patients with special needs: a survey of U.S. programs. J Dent Educ 72:1010–1019 18768443

[44] Krause L, Seeling S, Prütz F, Wager J (2022) Toothache, tooth brushing frequency and dental check-ups in children and adolescents with and without disabilities. J Health Monit 7:48–60 35434500 10.25646/9565 PMC9009068

[45] Huebner CE, Chi DL, Masterson E, Milgrom P (2015) Preventive dental health care experiences of preschool-age children with special health care needs. Spec Care Dentist 35:68–77 25082666 10.1111/scd.12084 PMC4312543

[46] Boyle CA, Boulet S, Schieve LA, Cohen RA, Blumberg SJ, Yeargin-Allsopp M, Visser S, Kogan MD (2011) Trends in the prevalence of developmental disabilities in US children, 1997–2008. Pediatrics 127:1034–1042 21606152 10.1542/peds.2010-2989

[47] Tesini DA (1981) An annotated review of the literature of dental caries and periodontal disease in mentally retarded individuals. Spec Care Dentist 1:75–87 6454266 10.1111/j.1754-4505.1981.tb01232.x

[48] Wyne AH (2002) Dental management of mentally retarded patients. Pakistan Oral Dent J 22:3–8

[49] Moldvai J, Orsós M, Herczeg E, Uhrin E, Kivovics M, Németh O (2022) Oral health status and its associated factors among post-stroke inpatients: a cross-sectional study in Hungary. BMC Oral Health 22:234 35701775 10.1186/s12903-022-02259-2 PMC9195382

[50] Orsós M, Moldvai J, Németh O (2019) [Oral health of people with special needs] [Hungarian]. Fogorv Szle 112:59–61

[51] Orsós M, Moldvai J, Simon F, Putz M, Merész G, Németh O (2021) Oral health status of physically disabled inpatients. Results from a Hungarian single-centre cross-sectional study. Oral Health Prev Dent 19:699–706 34918504 10.3290/j.ohpd.b2448609 PMC11640792

[52] Moldvai J, Orsós MM, Simon F, Merész G, Németh O (2019) Descriptive study of oral health, dental care and health behavior of inpatients undergoing physical medicine and rehabilitation. Oral Health Care 4:1–4

[53] Abullais SS, Al-Shahrani FM, Al-Gafel KM et al (2020) The knowledge, attitude and practices of the caregivers about oral health care, at centers for intellectually disabled, in Southern region of Saudi Arabia. Healthcare (Basel) 8:416 33096596 10.3390/healthcare8040416 PMC7712856

[54] Zhou N, Wong HM, McGrath C (2020) Toothbrush deterioration and parents’ suggestions to improve the design of toothbrushes used by children with special care needs. BMC Pediatr 20:443 32958022 10.1186/s12887-020-02347-8 PMC7504597

